# Polycystic Ovary Syndrome and Exercise: Evaluation of YouTube Videos

**DOI:** 10.7759/cureus.35093

**Published:** 2023-02-17

**Authors:** Ayhan Atigan, Alev Atigan

**Affiliations:** 1 Gynecology and Obstetrics, Karabük University, Medicine Faculty, Karabük, TUR; 2 Physical Medicine and Rehabilitation, Karabük University, Karabük, TUR

**Keywords:** gqs, vpi, discern, insulin resistance, diet, exercise, pcos, youtube

## Abstract

Objectives: Polycystic ovary syndrome (PCOS) is a common endocrine disorder in the reproductive female population. These young patients often and easily watch YouTube videos on the Internet to learn about their condition and find a natural solution. Our goal is to analyze the contents of PCOS exercise videos.

Methods: In July 2022, research data were collected by typing the term "PCOS exercise" in the search tab on the incognito YouTube page. One hundred and ninety eight videos that met the inclusion criteria were analyzed in detail. The basic data of the videos available on YouTube was recorded. In addition, the DISCERN, global quality score (GQS), and video power index (VPI) scoring systems were calculated by two independent researchers.

Results: The profiles of the video uploaders were: health employee 28 (14.1%), nutritionist 25 (12.6%), sports trainer 48 (24.2%), patient 21 (10.6%), undefined 76 (38.4%), and their countries were: India 91 (46%), Europe and England 36 (18.2%), USA and Canada 54 (27.3%), and other countries 17 (8.6%). The distribution of video content was yoga 58 (29.3%), aerobic exercise 38 (19.2%), strengthening exercise 44 (22.2%), and unified 58 (29.3%). The mean values were: video duration (15.27±11.27), total views (3,070,957±16,474,197), likes (48,116±283,308), dislikes (930±4102), VPI (97.82±7.28), GQS (3.89±1.05), DISCERN (33.62±10.42), subscriber counts (985,614±2,222,354), and comment counts (1741±10,689). Europe-England and America-Canada videos were of better quality for DISCERN and GQS scores than those from other countries.

Conclusion: Overcoming PCOS requires a lifestyle change, including exercise and diet. There is no consensus on which type of exercise is better yet. However, the necessity of regular exercise is known. We showed yoga and Indian hegemony in YouTube "PCOS exercise" videos.

## Introduction

Polycystic ovary syndrome (PCOS) was defined by Stein and Leventhal in 1935 [1]. PCOS is seen in prevalences ranging from 4% to 26% [2]. It can affect the mental and physical health, menstrual pattern, and fertility of these women in reproductive age [3]. The Rotterdam criteria including oligo-anovulation, hyperandrogenism, and the appearance of polycystic ovaries are helpful in diagnosis. Although there is no cure at present, symptomatic treatments are available.

PCOS has metabolic and cardiovascular effects. It is observed that in the distribution of type 2 diabetes mellitus cases, PCOS patients are 4-5 times more common than those without PCOS [4]. Despite the fact that the exact causes cannot be revealed, there are hormonal imbalances and insulin resistance [3]. Whether underweight or overweight, PCOS patients have intrinsic insulin resistance. As the patient's weight increases, the severity of the resistance may also increase [5]. The increase in obesity, the increase in stress in daily life, and the decrease in physical activity are the leading causes. Although there are drugs to break insulin resistance, these drugs do not provide long-term benefits unless the root cause of the problem is resolved. Modulation of lifestyle by exercising regularly, increasing physical activity in daily life, and consuming conscious foods reduces insulin resistance. It can be ensured that the body remains in shape by losing weight with routine exercises.

Physical activity that increases the body's energy consumption is called exercise when done regularly and repetitively to maintain health or increase physical condition. The basis of the exercise is the number of repetitions, duration, intensity, applicability, and type. Although there are many classifications, stretching (yoga), aerobic, and strengthening exercises can be mentioned as the main exercise types [6].

YouTube is a huge social media platform where millions of videos are uploaded, and so many videos are clicked tens of times almost every day [7]. Even if most people go to the doctor for their complaints, they search for something about their disease on the internet. These researches are mostly carried out on Youtube because it contains visuals and is easily accessible. On this platform, which reflects society on the internet, there is very useful information as well as garbage-value information. Our goal is to analyze these videos to reveal the quality of the video content.

## Materials and methods

Ethics committee approval was not required since publicly accessible information was used on youtube.com, a widely used social media platform, in accordance with the Declaration of Helsinki. This research was done on YouTube in July 2022. A search was made by typing "PCOS exercise" in the YouTube search bar in Google Chrome incognito tabs. Videos are sorted by relevance. All videos were evaluated by two independent, experienced researchers. A total of 627 videos were evaluated. A total of 429 videos were excluded, including 53 that were irrelevant, 6 that were repetitive, 63 that were non-English, 194 that had less than 1000 views, 105 YouTube-short videos, and 8 commercial advertisements.

A total of 198 videos were included in the study and analyzed statistically. Image type, uploader, country, content, upload time, duration, total views, likes, dislikes, number of subscribers, and number of comments were recorded. The exercise types suggested in the video content were divided into four categories: yoga, aerobics, strengthening, and unified, where multiple exercise types are recommended. During the pandemic, when spending more time at home, it was considered essential to upload videos. It was recorded that the narrator of the video talked about insulin resistance, fertility, menstrual patterns, and hormonal balance. Dietary advice and citation to a specific article were also considered.

Parameters showing video quality were calculated. The video power index (VPI) score was calculated with the formula [likes/(likes+ dislikes)] × 100 [8]. The DISCERN and global quality score (GQS) were calculated independently by each researcher, and their means were included in the statistics. DISCERN is a scoring system consisting of three sections and a total of 16 questions. In this scoring system, where high scores indicate quality, the scores vary between 1 and 5 points for each question. The first article of the DISCERN scoring system has over 1500 citations. [9]. Scoring for GQS ranges from 1 (poor quality) to 5 (excellent quality) [10].

Statistical analysis

The IBM SPSS Statistics (Version 21.0, SPSS, Inc.) program was used for the statistical analyses. Continuous variables were given as mean ± standard deviation, and categorical variables were given as numbers (%). The normal distribution of the data was examined by using the Kolmogorov-Smirnov test. The Mann-Whitney-U test and one-way ANOVA were used for comparisons between groups. p˂0.05 was considered significant in all statistical comparisons.

## Results

The main features of the videos are presented in Table 1. In the analysis of 198 videos included in the study, there were 5 (2.5%) videos with image-type animation, while 193 (97.5%) videos contained real images. Video narrators include sports trainers 48 (24.2%), health employees 28 (14.1%), nutritionists 25 (12.6%), patients 21 (10.6%), and 76 unidentified (38.4%) groups. When a YouTuber's country was examined, India was at 91 (46%), European countries and England (UK) were at 36 (18.2%), the United States of America (USA) and Canada were at 54 (27.3%), and other countries were at 17 (8.6%). In the examination of video exercise contents, yoga 58 (29.3%), aerobic exercise 38 (19.2%), strength training 44 (22.2%), and unified exercise 58 (29.3%), consisting of a combination of at least two of these exercises, were observed. Most of the videos (133-67.2%) were uploaded after the pandemic. Parameters highlighted along with exercise in PCOS exercise videos were: insulin resistance 71 (35.9%), fertility and/or infertility 31 (15.7%), menstrual regulation and/or irregularity 52 (26.3%), hormonal balance and/or imbalance 85 (42.9%), dietary recommendation 74 (37.4%), and citing a scientific article 17 (8.6%).

**Table 1:** Distrubition of video type

Features			N	%
Image type		Real	193	97.5
		Animation	5	2.5
Uploaders		Health employee	28	14.1
		Nutritionist	25	12.6
		Sports trainer	48	24.2
		Patient	21	10.6
		Unidentified	76	38.4
Country group		India	91	46
		Europe and England (UK)	36	18.2
		USA and Canada	54	27.3
		Other countries	17	8.6
Video content		Yoga	58	29.3
		Aerobic exercise	38	19.2
		Strengthening exercise	44	22.2
		Unified	58	29.3
Loading time		Pre-pandemic	65	32.8
		Post-pandemic	133	67.2
Highlighted in the video	Insulin resistance	Yes	71	35.9
		No	127	64.1
	Fertility/Infertility	Yes	31	15.7
		No	167	84.3
	Menstrual regulation/irregulation	Yes	52	26.3
		No	146	73.7
	Hormone balance/imbalance	Yes	85	42.9
		No	113	57.1
	Diet advice	Yes	74	37.4
		No	124	62.6
	Scientific article cited	Yes	17	8.6
		No	181	91.4

Descriptive information on video scores is shown in Table 2. The video duration mean value is 15.27±11.27 minutes. Videos had a mean value of 3,070,957±16,474,197 views, 48116±283,308 likes, 930±4102 dislikes, 985,614±2,222,354 subscriber counts, 1741±10,689 comment counts. The mean values of the scales showing the video quality were VPI 97.82±7.28, GQS 3.891.05, and DISCERN 33.62±10.42.

**Table 2:** Analysis of video scores VPI: Video Power Index, GQS: Global Quality Score

Features	Mean	Std deviation	Median	Min	Max
Video duration, minutes	15.27	11.27	12.46	1.09	64.05
Total views	3,070,957	16,474,197	109,407	1108	195,244,880
Likes	48,116	283,308	2714	0	3,860,599
Dislikes	930	4102	16	0	39,494
VPI	97.82	7.28	98.74	0	100
GQS	3.89	1.05	4	1	5
DISCERN	33.62	10.42	32	16	70
Subscriber counts	985614	2,222,354	150,000	171	24,000,000
Comment counts	1741	10,689	194	0	147,240

**Table 3:** Analysis of video quality indicators VPI: Video Power Index, GQS: Global Quality Score

Video source		N	Views mean ± Std	p-value	DISCERN mean ± Std	p-value	GQS mean ±Std	p-value	VPI mean ± Std	p-value
Video uploader										
Professional		101	4,094,051±57,629	0.374	35.85±10.42	0.002	4.03±0.94	0.065	98.12±2.47	0.555
Non-professional		97	2,005,674±7,598,405		31.29±9.95		3.75±1.14		97.51±10.11	
Video release date										
Pre-pandemic		65	6,028,906±27,075,433	0.200	33.31±10.02	0.772	3.85±0.95	0.657	98.45±1.68	0.394
Post-pandemic		133	1,625,343±6,572,393		33.77±10.64		3.92±1.10		97.51±8.80	
Country group										
India		91	864,783±1,780,249	0.06	33.71±11.28	0.302	3.80±1.12	0.007	98.54±1.42	0.187
Europe-England(UK)		36	1,801,197±4,082,255		34.17±7.75		4.31±0.82		98.44±1.30	
USA-Canada		54	4,858,268±16,745,900		34.50±10.67		3.96±0.88		95.97±13.60	
Other countries		17	11,892,038±47261469		29.12±9.32		3.29±1.26		98.52±3.03	
Exercise type										
Yoga		58	504,409±712,287	0.256	33.14±9.54	0.950	3.95±0.92	0.570	96.92±13.01	0.688
Aerobic		38	3,829,009±16,555,827		33.45±11.59		3.89±1.15		97.78±2.45	
Strengthening		44	1,564,757±3,018,290		34.36±9.83		4.02±0.90		98.10±2.70	
Unified		58	6,283,485±27,080,504		33.64±11.11		3.74±1.20		98.53±1.80	
Subscriber count	Less	152	565,086±2,046,800	0.031	32.99±10.35	0.123	3.79±1.09	0.011	97.81±8.23	0.980
	More	46	11,351,226±32,905,481		35.70±10.50		4.24±0.82		97.84±2.19	
Insulin resistance	Yes	71	417,727±925,620	0.024	38.73±12.70	0.000	4.18±1.00	0.004	98.50±2.26	0.324
	No	127	4,554,259±20,436,977		30.76±7.56		3.73±1.05		97.44±8.92	
Fertility/Infertility	Yes	31	580,294±1,303,784	0.361	41.29±14.07	0.001	4.39±0.88	0.004	98.62±1.50	0.507
	No	167	3,533,296±17,899,761		32.19±8.95		3.80±1.06		97.67±7.90	
Menstrual regulation/irregulation	Yes	52	558,042±1,130,339	0.034	41.29±12.16	0.000	4.33±0.76	0.000	98.59±1.32	0.377
	No	146	3,965,968±19,110,415		30.88±8.18		3.74±1.10		97.55±8.43	
Hormone balance/imbalance	Yes	85	442,173±930,782	0.026	37.42±12.24	0.000	4.08±0.96	0.026	98.46±2.20	0.282
	No	113	5,048,361±21,622,497		30.75±7.70		3.75±1.09		97.33±9.44	
Diet advice	Yes	74	3,230,005±22,654,830	0.917	38.20±13.07	0.000	4.00±1.13	0.275	98.65±1.42	0.217
	No	124	2,976,042±11,403,904		30.88±7.23		3.83±1.05		97.32±9.11	
Scientific article cited	Yes	17	700,049±1,584,454	0.536	45.82±14.95	0.002	4.53±0.87	0.009	98.24±1.98	0.803
	No	181	3,293,639±17,211,258		32.47±9.14		3.83±1.05		97.78±7.59	

Scales showing video quality were compared in Table 3. A professional group (n=101) including health employees, sports trainers, and nutritionists was created. A non-professional group (n=97) was made with the patients and the unidentified. While all video quality indicators were higher in the professional group, only DISCERN was statistically significantly different (35.85±10.42 vs 31.29±9.95, p=0.002). Although the number of videos created in the post-pandemic (n=133) period was twice as high as before (n=65), there was no statistical difference between the quality indicators of video content (p>0.05 for DISCERN, GQS, and VPI). Videos originating from India (n=91) were almost equal to the sum of USA-Canada and Europe-UK videos (n=90). There was a statistically significant difference only for GQS when comparing country groups with an ANOVA (p=0.007). In addition, the Europe-UK group had a significantly higher score. There was no difference between the groups in the quality analysis of the videos according to the exercise type (p>0.05). Two groups were formed according to whether the number of subscribers was more or less than the mean subscribers. As the number of subscribers on YouTube increased, the number of views and the GQS score were also increasing (p=0.031 and p=0.011, respectively). DISCERN and VPI are unaffected by subscriber count (p>0.05). In addition to the exercise content of the video, the number of views was significantly lower in the videos that emphasized the scientific data of PCOS, while the video quality scores were found to be statistically significantly higher.

## Discussion

Millions of clicks are made on YouTube every day [7]. On YouTube, which serves as a consultant doctor, patients go to do in-depth research on something they have heard about their health. People strive to take the necessary care for their personal development and health. Especially during the time spent at home due to the COVID pandemic, home-based exercises have become even more popular to protect physical health [11]. Thus, both the number of videos and the number of views increased. Our study found that the number of videos doubled after the pandemic. In fact, we have seen that there are not enough animation videos about exercise, which is an area where animation videos can be easily published. While the DISCERN scores of the videos of the professional video uploaders were statistically significantly higher, there was no difference in other scores compared to the non-professional ones. Professionals using more scientific language can be effective in this regard.

PCOS workout videos could be called a crowded Indian family. Although there was no difference between the DISCERN and VPI scores, we showed that the Europe-UK-sourced videos were of good quality according to the GQS score. The Europe-UK and USA-Canada videos were above the mean value for DISCERN and GQS. In studies conducted in India, the prevalence of PCOS was found to vary between 3.7% and 22.5% [12]. Despite the prevalence of PCOS being similar to other countries, we found that Indian YouTubers are more interested in PCOS exercise video sharing than other countries. In addition, there are articles in the literature that argue that there is a rapid increase in prevalence, especially due to the increase in obesity [13,14]. One of the reasons why both Indian and yoga videos are in the majority, which we found in our study, might be the fact that yoga originated in India [15].

We have mentioned above that there are three (stretching, aerobic, and strengthening) main types of exercise [6]. A traditional method that is a good fighter against stress is yoga. We can say that yoga videos take the lead in the analysis of the videos. Yoga can be classified as a stretching exercise [16]. Yoga regulates blood circulation through breathing and mind exercises. Types of cardiovascular movement such as brisk walking, swimming, running, and cycling that increase heart rate and breathing are called aerobic exercise. Lifting weights, working with resistance bands, and other movements that can be done by yourself are called strengthening exercises. The videos in which these exercises were suggested in combination were quite common. However, a clear exercise has not yet been recommended for PCOS [17], and there was no statistically significant difference in the quality of the PCOS exercise videos in our study.

The long-term effects of PCOS include serious diseases such as heart disease, diabetes, hypertension, infertility, and cancer. Insulin resistance has been reported in almost all patients diagnosed with the Rotterdam diagnosis [5]. Insulin resistance gets worse as BMI increases. Obesity in women brings about changes in the endocrine system and blood androgen levels [18]. When this cycle is broken in a positive way, a regression in PCOS symptoms is observed. Although the benefits of regular exercise on PCOS are known, there is no consensus on the type of exercise that is optimal [19]. Regular aerobic exercise helps to lose weight and regulates glucose levels [20].

A good exercise program should be supported by conscious food consumption. The number of views of the videos in which diet advice was given along with exercise was high. DISCERN, GQS, and VPI scores were higher for those with recommended diets, but statistically significantly different for DISCERN. A calorie-restricted diet with a low glycemic index decreased testosterone and insulin resistance levels. An anti-inflammatory, low-glycemic-index, low-fat diet has positive effects on PCOS symptoms [21]. Although a calorie-restricted and low-fat diet style was recommended in the videos we observed in our study, statistical analysis was not performed.

The characteristic features of PCOS patients are a sedentary life with reduced physical activity, chronic hormonal imbalance, unconscious eating habits, and menstrual irregularity. Although the number of views and the VPI of the videos describing these features were low, the DISCERN and GQS scores were statistically significantly higher in our study. There is an increase in testosterone, dehydroepiandrosterone, and its sulphate ester (DHEAS) levels, especially in obese women with PCOS [18]. Excessive androgen production occurs when the concentration of luteinizing hormone (LH) rises relative to follicle-stimulating hormone (FSH), which is more common in women with PCOS [21]. Chronic hormonal imbalance is manifested by numerous cysts and irregular menses. Anovulatory cycles are one of the important causes of female infertility. Approximately one in eight women has fertility problems. 25% of women with fertility problems have trouble with ovulation [22]. Prolonged exercise during the day can also cause anovulation. However, when daily exercise is kept up for 30 to 60 minutes, it reduces the risk of anovulation-induced infertility [23]. One review stated that while aerobic exercise had no effect on LH, yoga reduced LH levels [24].

The lack of detailed analysis of diet programs, which are an integral part of the exercise, is one of the significant limitations of this study.

## Conclusions

PCOS is a common endocrine disorder in which symptoms can regress with exercise. When we saw the application of the videos, we showed that there is no excuse to avoid exercise and that it can be easily applied at home. People who are away from stress, have regular sleep, pay attention to their diet, and exercise regularly are one step ahead of PCOS. We believe that our study will contribute to future studies on PCOS, which still has a great dilemma about its cause, treatment, and even which type of exercise is better.
